# Asymmetric Sensory-Motor Regeneration of Transected Peripheral Nerves Using Molecular Guidance Cues

**DOI:** 10.1038/s41598-017-14331-x

**Published:** 2017-10-30

**Authors:** Sanjay Anand, Vidhi Desai, Nesreen Alsmadi, Aswini Kanneganti, Dianna Huyen-Tram Nguyen, Martin Tran, Lokesh Patil, Srikanth Vasudevan, Cancan Xu, Yi Hong, Jonathan Cheng, Edward Keefer, Mario I. Romero-Ortega

**Affiliations:** 1 Department of Bioengineering, University of Texas at Dallas, 800 W. Campbell Road, Richardson, TX 75080 USA; 2 Department of Bioengineering, University of Texas at Arlington, 500 UTA Blvd, Arlington, Texas 76010 USA; 3 Department of Plastic Surgery, University of Texas Southwestern Medical Center, 5323 Harry Hines Blvd., Dallas, TX 75390 USA; 4 Nerves Incorporated, P.O. Box 141295 Dallas, TX 75214 USA; 5 Division of Biomedical Physics, Office of Science and Engineering Laboratories, Center for Devices and Radiological Health, U.S. Food and Drug Administration, Silver Spring, MD 20993 USA

## Abstract

Neural interfaces are designed to decode motor intent and evoke sensory precepts in amputees. In peripheral nerves, recording movement intent is challenging because motor axons are only a small fraction compared to sensory fibers and are heterogeneously mixed particularly at proximal levels. We previously reported that pain and myelinated axons regenerating through a Y-shaped nerve guide with sealed ends, can be modulated by luminar release of nerve growth factor (NGF) and neurotrophin-3 (NT-3), respectively. Here, we evaluate the differential potency of NGF, glial cell line-derived neurotrophic factor (GDNF), brain-derived neurotrophic factor (BDNF), pleiotrophin (PTN), and NT-3 in asymmetrically guiding the regeneration of sensory and motor neurons. We report that, in the absence of distal target organs, molecular guidance cues can mediate the growth of electrically conductive fascicles with normal microanatomy. Compared to Y-tube compartments with bovine serum albumin (BSA), GDNF and NGF increased the motor and sensory axon content, respectively. In addition, the sensory to motor ratio was significantly increased by PTN (12.7:1) when compared to a BDNF + GDNF choice. The differential content of motor and sensory axons modulated by selective guidance cues may provide a strategy to better define axon types in peripheral nerve interfaces.

## Introduction

Prosthetic devices have advanced from traditional mechanical hooks performing simple open/close tasks to anthropomorphic robotic hands capable of complex movements with up to 22 degrees of freedom and equipped with multiple sensors and embedded controllers for implementing automatic grasp and providing sensory feedback^1,2^. Despite such progress, current prostheses are controlled through surface electromyography (EMG) signals and are operated by visual or surrogate sensory feedback which complicates the use of the robotic limbs and contributes to the eventual abandonment of these devices due to lack of embodiment^3^. Decoding motor intent for robotic limb control and conveying specific sensory modalities from the electronic skin to the user have been proposed as viable alternatives. Cortical interfaces in individuals with spinal cord injury have shown great promise in achieving volitional control of a prosthetic limb and eliciting sensory percepts through microstimulation of the sensory cortex^4–6^. However, for limb amputees, peripheral nerve stimulation offers a less invasive alternative to cortical interfaces and direct access to functional motor and sensory pathways in the residual limb^7^.

Several electrode configurations have been developed to interface with peripheral nerves, including extraneural cuffs, intrafascicular electrode arrays, and regenerative based electrodes^8,9^. The external cuff and intrafascicular electrodes have been used successfully to elicit sensory feedback as well as recording motor intent in amputees. Typical precepts elicited include digit flexion, constant pressure, natural tapping, and vibration. Variations in stimulus parameters such as pulse width, amplitude, and frequency modulate the percept type and quality^10–13^. However, patients also perceived abnormal paresthesia including tingling and burning sensations^10,12,14^, which has been related to indiscriminate electrical depolarization of multiple sensory modalities, including pain and temperature C-fibers, by the different electrodes^15^. This is due to the fact that axons of similar biophysical characteristics such as the large myelinated proprioceptive and mechanoreceptive fibers or medium myelinated slow and rapidly adapting mechanoreceptors have overlapping depolarizing thresholds^16^. Furthermore, recording selectively from motor axons in somatic nerves is challenging because of the small number of these fibers compared to sensory axons and their heterogeneous distribution^17^.

The ability of transected nerves to regrow through multi-electrode arrays forming regenerative interfaces was demonstrated more than 20 years ago^18^. Using this approach, we have shown that peripheral nerves can be directed to grow through an 18-electrode array placed in the lumen of a conduit^19,20^. This regenerative multi-electrode interface (REMI) records single units as early as 7 days from both motor and sensory sub-modality axons from animals in which the interfaced nerves are allowed to reinnervate their original target in the skin and muscle^21^. Given the critical role of neurotrophic factors (NTFs) from Schwann cells, muscle and skin targets, both during early development and post-injury in the adult peripheral nervous system (PNS)^22–24^, we have proposed that such guidance cues can be used in regenerative neural interfaces to influence the selective interaction of motor and sensory subtype-axons with distinct electrodes.

Motor axons follow chemoattractant gradients of glial cell line-derived neurotrophic factor (GDNF), through tyrosine kinase Ret and GFRα2 receptors^25^. They also integrate membrane bound signals like Celsr3/cadherin in their path toward muscle targets^26,27^. Conversely, pain and temperature related sensory fibers are attracted by gradients of nerve growth factor (NGF), mechanoceptive neurons are influenced by brain-derived neurotrophic factor (BDNF), and neurotrophin- 3 (NT-3) is critical for reinnervation of proprioceptive neurons to muscle spindles^28–32^. Furthermore, adding NGF to injured nerves can selectively guide the regeneration of TrkA nociceptive neurons^33,34^, while NT-3 guides TrkC + proprioceptors^35–37^, and BDNF is known to stimulate TrkB-mechanoceptive fibers^38^. Pleiotrophin (PTN), another neurotrophic factor, is also reported to be expressed in ventral roots after injury and promotes spinal motor neuron regeneration^39^. Indeed, in the adult PNS, exogenous NGF effectively guides the regeneration of nociceptive axons into the dorsal spinal cord after dorsal rhizotomy^33^ and doubles the ratio of sensory to motor (S/M) axons innervating the NGF-expressing branch in the femoral nerve bifurcation injury model^34^.

We have previously reported that compartmentalized delivery of NGF and NT-3 modulates the regeneration of nociceptive and large myelinated axons in dorsal root ganglion (DRG) cultures and amputated peripheral nerves *in vivo* ^40^. Here, we evaluated the effect of single and combined NTFs in selectively guiding sensory and motor neurons into separate and closed compartments of a Y-shaped conduit after nerve transection. First, the effect of individual NTFs was evaluated against that of BSA, a neutral cue, in the other compartment of a Y-shaped conduit. Second, an *in vivo* assay was used to evaluate the pathway favored by regenerating axons when presented with competing choices in the form of different NTFs loaded in opposite compartments. The results demonstrate that GDNF had a stronger effect than PTN, NT-3, BDNF, and NGF to attract motor axons compared to BSA. Furthermore, the sensory-to-motor (S/M) ratio of regenerated axons was significantly increased in the PTN loaded side of the Y-conduit compared to the BDNF + GDNF compartment.

## Material and Methods

### Microencapsulation of Neurotrophic Factors (NTFs)

**Figure 1:**
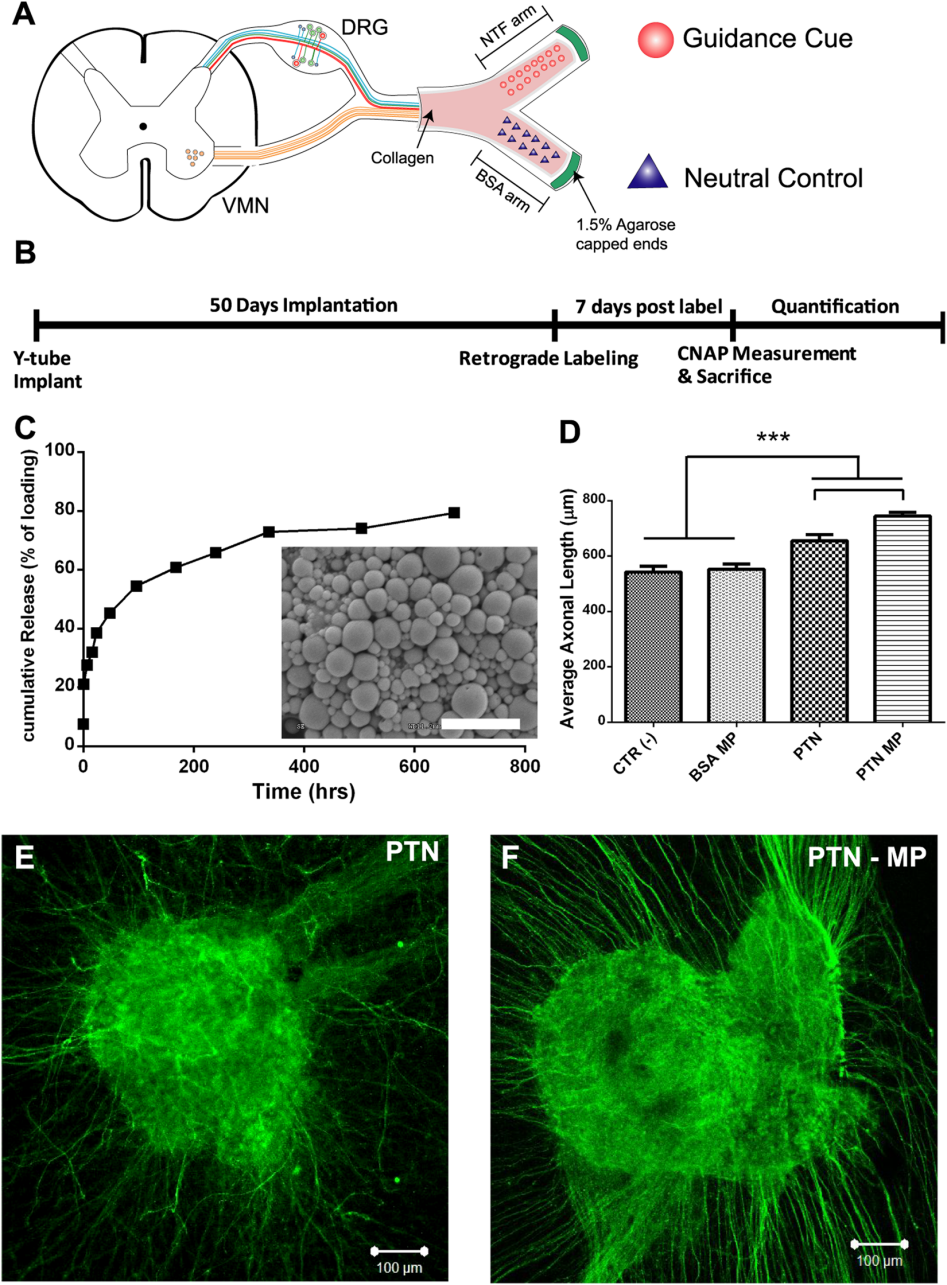
Experimental design. (**A**) Schematic of Y-conduit containing microparticles with a neurotrophic factor in one compartment and BSA neutral control in the other. The transected mixed sciatic nerve is placed in the common arm of the Y-conduit and distal ends are capped with 1.5% agarose. (**B**) Timeline of the experimental procedures following implantation. (**C**) PTN cumulative release curve over 30 day period and scanning electron microscope of PTN-MP with size ranging from 0.3–1.5 µm (inset; Scale bar = 3 µm). (**D**) Compared to control and BSA MP, the average axonal length in neonatal DRGs for PTN and PTN MP was significantly higher (N = 3/group; One-way ANOVA; F (3, 263) = 33.52; R^2^ = 0.2766; ***indicates P ≤ 0.001). Data represented at mean ± SEM. (**E**,**F**) Representative images of DRGs exposed to PTN and PTN-MP.

Biodegradable microparticles (MPs) were made with poly(DL-lactic-co-glycolic acid) (PLGA) using double emulsion as reported previously^41^. Briefly, PLGA 50:50 (Lakeshore Biomaterial, St. Louis, MO) was dissolved in dichloromethane (DCM) 200 mg/ml (Sigma-Aldrich, St. Louis, MO), and mixed with aqueous solutions of human recombinant NGF (7 S, 13.5 kD; Invitrogen), BDNF (27 kD), NT-3 (13.6kD), GDNF (15 kD), or PTN (15.4 kD) (20 μg/ml; Prepotech Inc, NJ) or BSA (20 μg/ml; Sigma-Aldrich, St. Louis, MO). The solutions were added to polyvinyl alcohol (20 mg/ml) and emulsified. The resulting MPs were freeze-dried for 48 hours and stored at −20 °C. Loading efficacy was calculated at 67 ± 5% from release studies. The particles were evaluated by scanning electron microscope (SEM, Hitachi S-3000 N), and their size distribution was estimated at 800 nm wavelength using a Zeta Potential Analyzer. Released NGF and PTN into phosphate buffer saline solution (PBS) from the MPs were evaluated at 37 °C in a shaker incubator at hourly intervals, daily for a week, and weekly after that for 4 weeks, and quantified by ELISA (PTN; TSZ ELISA, HU9951) (Fig. 1B). BSA release was quantified using the BCA assay (Thermo Scientific, Rockford, IL) and read at 562 nm.

### DRG Bioactivity Assay

Neonatal (P0 - P4) mice (CD1) were used to obtain dorsal root ganglia (DRGs) and collected in Leibovitz’s L-15 Medium (Sigma-Aldrich, St. Louis, MO). The DRGs were cleaned of connective tissue and placed in poly-D-lysine (PDL) coated glass-bottom wells suspended in 10 μl of atelomeric chicken collagen (85% type I, 15% type II; Millipore; Temecula, CA). The explants were incubated at 37 °C with 5% CO_2_ for 15 minutes to allow gelation before adding 200 μl of Neurobasal A media (Sigma-Aldrich, St. Louis, MO) supplemented with 2% B27, 0.5% penicillin/streptomycin, and 0.75% L-glutamine. NTF MPs were compared to recombinant proteins at previously reported physiological concentrations: NGF, BDNF and PTN were tested at a 100 ng/ml, GDNF at 50 ng/ml and NT-3 at 5 ng/ml. The NTFs and NTF-MPs were added 24 hours after plating the DRGs. Control DRG explants were incubated in BSA-MPs. After 5 days in culture, the DRGs were fixed for 15 minutes in 4% PFA, rinsed and stored at 4 °C.

### DRG *in vitro* Immunocytochemistry

Fixed DRGs were permeabilized in 0.5% PBS-Triton ×100 for 5 minutes. Non-specific staining was blocked with 4% normal donkey serum for 1 hour, followed by incubation with a mouse anti-β tubulin III antibody (1:400; Sigma-Aldrich, St. Louis, MO) overnight at 4 °C. After rinsing, the tissue was incubated for 1 hour with a Cy2-conjugated donkey anti mouse antibody (1:400; Sigma-Aldrich, St. Louis, MO). The stained tissue was imaged on a Zeiss confocal microscope (Zeiss Axioplan 2 LSM 510 META). Axonal growth was estimated from 3 DRG samples per treatment. Z-stacks were imaged at 20X magnification (20 images each at 15.4 μm slice thickness) and individual axons were traced using ImageJ software measuring from the edge of the DRGs to the axon terminals (Fig. 1D–F).

### Y-tube Conduits

Poly(ester urethane) was synthesized from polycaprolactone, hexamethylene diisocyanate, and putrescine, according to reference^42^. The poly(ester urethane) Y-shaped tubes were made using Y-shaped molds made with dental wax (Polysciences Inc., Warrington, PA). The molds were dip coated 20–30 times to achieve a wall thickness of approximate 0.25 mm in 5% polyurethane/hexafluoro isopropanol solution. The coated tubes were dried overnight at room temperature, and the dental wax was removed by immersion in hexane. The Y-tube then was dried in air at room temperature. The Y-conduit common arm measured 5 mm and each of the two compartments measured 5–7 mm from the bifurcation point, with 1.5 mm ID and total length of 10–12 mm.

Y-tube conduits were disinfected with 70% ethanol followed by UV light irradiation. Collagen type I/III (EMD Millipore, Billerica, MA) was then used to fill the lumen of the Y-conduit. The common arm was filled with collagen using a 28-gauge insulin syringe up to the bifurcation and allowed to polymerize at 37 °C for 10 minutes. The “Y” compartments were then filled with 10 μL of NTF-MP or BSA-MP, mixed with collagen, and polymerized at 37 °C for 10 minutes before implantation.

### Modeling Release of Neurotrophic Factors in Y-shaped conduits

**Figure 2:**
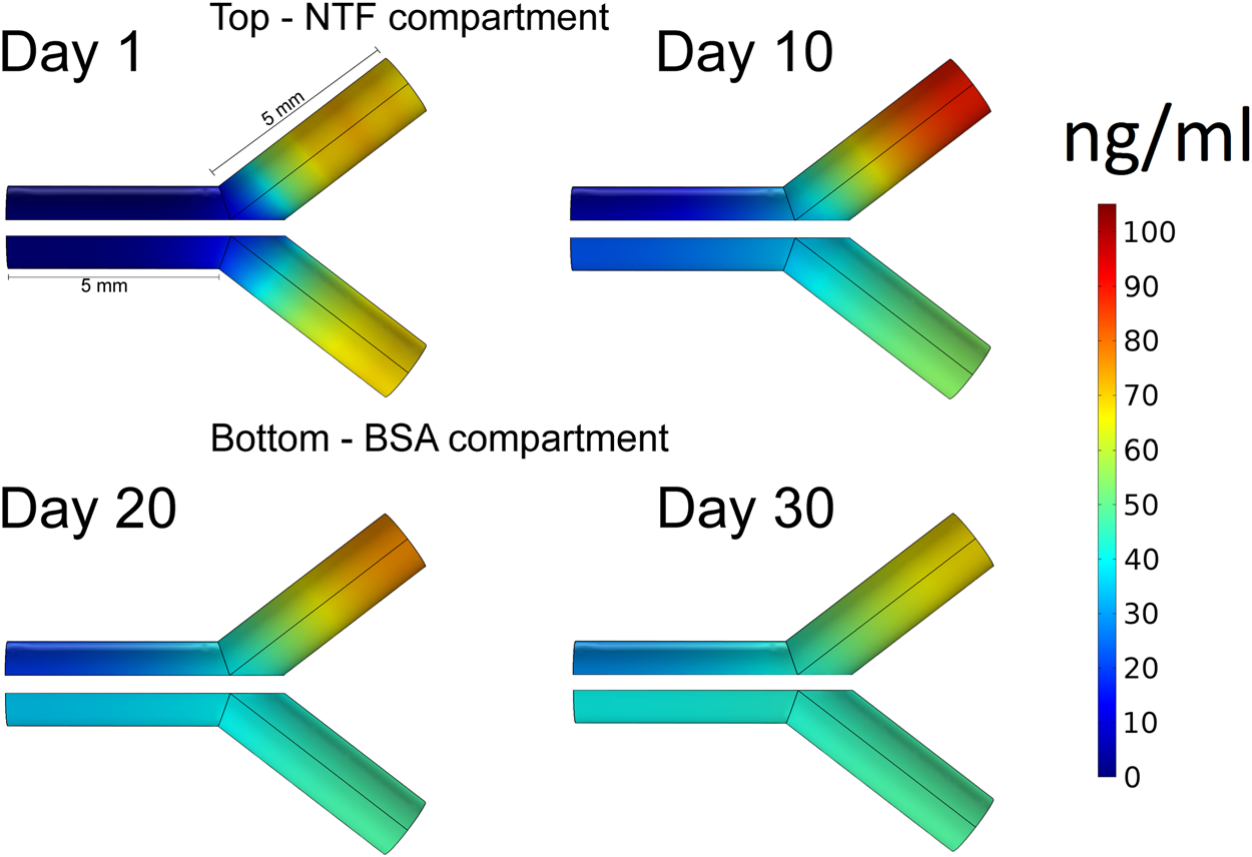
Diffusion of PLGA microparticles loaded with NTF and BSA over a 30 day time period. At Day 1 there is a burst release followed by a gradient formation in the subsequent days. The top compartment represents PTN-MP release and BSA-MP release in the bottom compartment. Arrow heads indicate the approximate position of nerve regeneration.

We estimated the amount of NTF-MPs needed to provide sustained release for 30 days in each compartment of the Y-conduit by modeling the protein release and diffusion of the NTF-MP as previously reported^43^. Briefly, finite element analysis (COMSOL, Inc.) was used to model the NTF concentration and diffusion in the collagen-filled lumen. One compartment of the Y-conduit was modeled for PTN release and another for BSA, according to the parameters specified in Table 1. The model considers 2 µm diameter PLGA-MP, and assumes no degradation of collagen, and isotropic protein diffusion. Using this model, we confirmed that separate chemotactic gradients could be established in both compartments of the Y-conduits in the first 10 days (Fig. 2).

**Table 1:** Dimension and diffusivity values for protein release from PLGA microparticles in collagen.

Parameter	Value	Description
D_PTN_	7.6 × 10^−12^ m2/s^43^	Diffusivity of PTN in 0.1% collagen
D_BSA_	2.2 × 10^−11^ ^44^	Diffusivity of BSA in 0.1% collagen
ID	1.5 mm	Channel internal diameter
L	5 mm	Channel length
v_microchannel_	10 μl	Volume within conduit compartment
d_microparticle_	2 μm	Diameter of PLGA microparticles
T_total_	1.34 ng	Total amount of releasable PTN in the compartment

### Animal Implantation

Two separate cohorts of adult female Lewis rats were included in this study. In the first cohort (n = 36), we evaluated the effect of single molecular attractants in five experimental and one control groups (n = 6 each). Experimental groups had Y-conduits with NTF in one compartment (NGF, BDNF, NT-3, GDNF, and PTN) and BSA in the opposite side while control (+) group had mixed-nerve-cap in both compartments (Table 2). The nerve-cap was a 2 mm distal segment of the transected sciatic nerve ligated at one end and sutured to arms of the Y-conduit with the open end facing the lumen. The nerve-cap secretes multiple growth-factors and will have a non-specific effect on axonal regeneration. The sciatic nerve was used as the non-injured control to determine the baseline number of retrograde traced motor and sensory neurons. The second cohort of 60 rats was used to assess the dual molecular guidance in a Y-choice assay in two control and three experimental groups (n = 12 each): 1) Tibial (motor related) - sural (sensory) nerve-cap targets (positive control), 2) Muscle (motor-related) - Skin (sensory) tissue-cap targets (positive control), 3) BDNF + GDNF − BSA, 4) BDNF + GDNF − PTN, and 5) BDNF + GDNF − NGF.

**Table 2:** Experimental groups for single and combination neurotrophic factors in a Y-choice assay.

Cohort	Compartment 1	Compartment 2	Number of implants	Objective
1	BDNF	BSA	6	Effect of individual Neurotrophic factors against neutral guidance cue BSA
1	GDNF	BSA	6	
1	NT-3	BSA	6	
1	PTN	BSA	6	
1	NGF	BSA	6	
1	Nerve-cap	Nerve-cap	6	
2	BDNF + GDNF	BSA	12	Effect of combinatorial Neurotrophic factors in a Y-choice assay
2	BDNF + GDNF	PTN	12	
2	BDNF + GDNF	NGF	12	
2	Tibial	Sural	12	
2	Muscle-cap	Skin-cap	12	

The animals were anesthetized using isoflurane (2–2.5%) in 100% oxygen prior to surgical procedure. The sciatic nerve was exposed by muscle-sparing incision between the semitendinosus and the bicep femoris muscles and transected before the trifurcation as described in reference^45^. The distal portion of the nerve was removed to avoid trophic effect from the distal nerves and/or end targets. The sciatic nerve was then secured into the proximal arm of the Y-conduits using 9.0 nylon sutures. The distal ends of the Y-tube were capped by adding 1.5% agarose, placed under the muscle and closed using 4.0 silk suture. The skin was then closed using staples and topical antibiotic ointment was applied. All animals received antibiotic (cephazolin; 5 mg/kg, IM) and pain control (sustained release Buprenorphine; 0.1 mg/kg, SC) treatment post-surgery. All animal procedures were approved by the Institutional Animal Care and Use Committees (IACUC) of the University of Texas at Arlington and The University of Texas at Dallas, in accordance to the NIH Guide for the Care and Use of laboratory Animals.

### Retrograde Labeling of Motor and Sensory Neurons and Quantification

Retrograde labeling from the distal end of the fascicles that regenerated into each of the Y-conduit compartments was used to trace the axon sub-types to its origin in the spinal cord and DRG. Briefly, the Y-tube was re-exposed 45 days-post implantation. With the Y-tube in place, sterile Vaseline was used to make a reservoir at the distal end of the Y-tube arm and injected at the bifurcation site to prevent leakage of the retrograde label into the common arm. Thereafter, 5 µL of 4% fluorogold (FG, Fluorochrome LLC, Denver, CO) was added to one arm and 5 µL of 10% fluororuby (FR, Fluorochrome LLC, Denver, CO) to the other arm of the exposed nerve for 1 hour. The Y-tube was then placed back under the muscle and closed using resorbable sutures and the skin stapled. The skin surface was coated with antibiotic ointment and the animal was given antibiotics (cephazolin; 5 mg/kg, IM) and pain control (sustained release Buprenorphine; 0.1 mg/kg, SC) treatment post-surgery. The neuronal cell bodies of the regenerated axons were visualized in the ventral spinal cord and DRG. FG tracing labeled 953 ± 30.2 motor and 6914 ± 603.9 sensory axons in the non-injured sciatic control nerves, with an estimated tracing efficiency of 60% for VMNs and 66% for somatic DRG neurons from a total 1600 VMN and 10,500 DRG neurons respectively^46,47^. In contrast, FR labeling showed inefficient uptake compared to FG and therefore was omitted from the study.

### Compound Nerve Action Potential (CNAP) Measurements

Seven days following the retrograde labeling procedure, the implant site was surgically reopened while the animal was anesthetized. The sciatic nerve and its regenerated arms were exposed, carefully freed from surrounding connective tissue, and a Parafilm® tape was placed underneath the nerves to ensure electrical isolation. The sciatic nerve was gently placed on a bipolar hook microelectrode (FHC Inc., Bowdoin, ME) close to the pelvic foramen for stimulation. A second pair of hook electrodes was used to record evoked CNAP responses distally from the individual regenerated arms. Mineral oil covered the contact between the electrodes and the nerve. The sciatic nerve was stimulated with 30 µs wide biphasic pulses 2 Hz frequency for about 2 min duration using a A-M Systems (Sequim, WA) optically isolated instrument (model 2100). The CNAP from each regenerated arm of the Y-conduit was recorded in response to supra-maximal stimulation amplitude, i.e. three times the threshold to activate onset-response, in a bandwidth of 3–5000 Hz and a 20x gain preamplifier using Omniplex Data Acquisition System (Plexon Inc., Dallas, TX). For offline time-synchronization, a copy of the stimulation output was directly fed into the data acquisition system.

The recorded data was analyzed offline using MATLAB. A composite CNAP response was generated from individual responses of 200–300 stimulation pulses using the Stimuli-triggered Averaging (STA) process. Briefly, signals were extracted in time-windows including data 50 ms post and 15 ms prior to stimulus, overlapped and averaged to form a STA-CNAP waveform. Evoked peak responses were defined as an increase in amplitude larger than 10% from the baseline noise level which ranged from −0.003 to +0.003 mV. The peak latency was calculated by measuring duration between the positive phase of the stimulation pulse to the maximum amplitude of the individual peaks. The corresponding conduction velocities were obtained by dividing the distance (range of 15–25 mm) between the stimulating and recording electrodes over peak latencies. The area under curve for each peak was calculated from onset of the peak to the trough using the “trapz” inbuilt MATLAB’s mathematical function, which approximates the integral using the trapezoidal method.

### Animal perfusion and tissue preparation

Animals were sacrificed by overdose injection of sodium pentobarbital (120 mg/kg, IP) and transcardially perfused with 4% paraformaldehyde (PFA). The regenerated Y-nerve were harvested and post fixed in 4% PFA/2.5% glutaraldehyde in 0.1 M Cacodylate buffer for electron microscopy (EM). The spinal cord and the L4 and L5 Dorsal root ganglion were isolated, post fixed and cryoprotected in 30% sucrose prior to OCT embedding for cryosectioning. Sagittal sections were obtained at 20 μm thickness and DRG cross sections at 10 μm thickness. The sections were mounted onto glass slides serially and stored at −20 C until used.

### Quantification of retrograde labeled motor and sensory neurons

All slides were rinsed in PBS to remove the OCT and treated for lipofuscin reduction by incubating the tissue in 750 μM cupric sulfate/50 mM ammonium acetate for 40 minutes as reported elsewhere^48^. The tissue was then rinsed in PBS and cover-slipped using immuno-mount (Fisher Scientific, Waltham, MA). The FG+ and FR+ cells in the spinal cord and DRG were identified with a Zeiss fluorescence microscope using a wide band ultraviolet (UV) excitation filter. To prevent double counting, only positive retrograde labeled cells with a distinct nucleolus were included. Positively labeled DRG were also counted and the area was analyzed using the analyze-particle option of Image J, processing software. The DRG soma area was categorized into small (<300 μm^2^), medium (300–700 μm^2^), and large (≤700 μm^2^). Labeled cell quantification was corrected by accounting for section thickness and split nuclei count using methods described by Abercrombie^49^.

### Electron microscopy morphometry analysis

A subset of groups (nerve-cap control, GNDF, NGF, PTN, BDNF + GDNF and respective BSA compartments) were processed for EM (n = 3 animals per group; 3 random areas were imaged; 100–300 axons per area) to evaluate the axon type composition and myelination. The fixed tissue was embedded in resin and sectioned at 1 μm thickness using an ultra-microtome. The thin sections were stained with toluidine blue and photographed. Osmium stained sections were visualized using a JEOL LEM 1200 EX II microscope. Three fields of view per section with an area of 1520 µm^2^ were imaged (4000x magnification) and analyzed for the number of unmyelinated and myelinated axons, fiber (axon + myelin) diameter, axon diameter, and g-ratio using Image J software.

### Statistical analysis

The groups were compared using one-way ANOVA and Bonferroni’s ad-hoc multiple comparison test between the compartments of the Y-conduits using Prism 6 software (GraphPad Software Inc.). A p ≤ 0.05 was considered statistically significant. The data is presented as the mean ± standard error of the mean.

### Data availability

The data that support the findings of this study are available from the corresponding author upon reasonable request.

### Disclaimer

The mention of commercial products, their sources, or their use in connection with material reported herein is not to be construed as either an actual or implied endorsement of such products by the Department of Health and Human Services.

## Results

### Functional splitting of transected peripheral nerves

**Figure 3:**
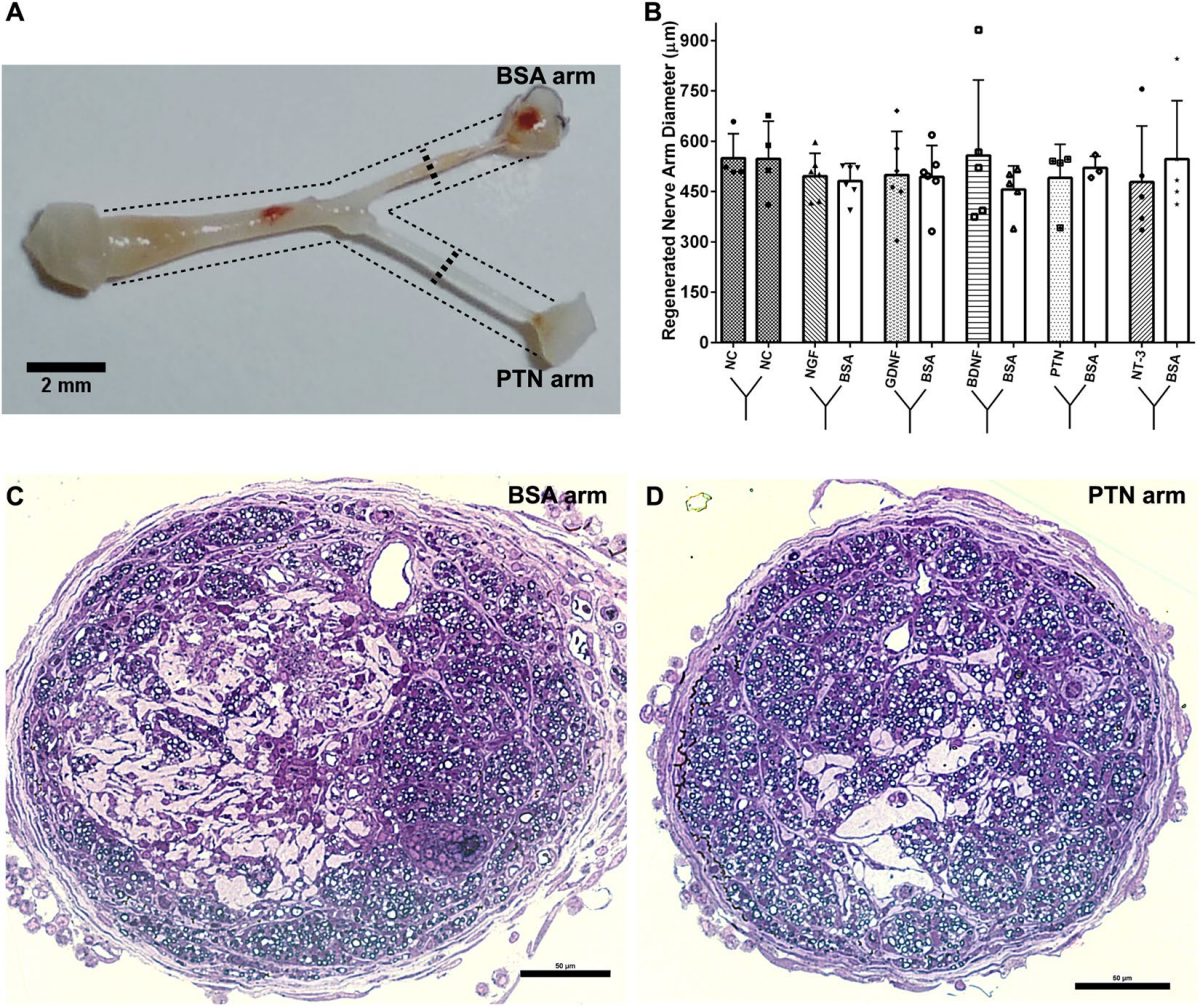
Comparable Y-split nerve regeneration using neurotrophic factor loaded microparticles (NTF-MP) with no distal targets. (**A**,**B**) Representative image of a regenerated Y-nerve and similar nerve fascicle diameter. (**C**,**D**) Toluidine stained semi thin sections for the BSA arm and PTN arm show perineurium and endoneurium formation with myelinated axons. Data represented as mean ± SEM; N = 3–6 animals/group.

Gross evaluation of the regenerated nerves into the Y-shaped conduits 45 days after implantation confirmed that the injured nerves extended along the 5 mm common arm, and bifurcated into two similar size branches (4–5 mm long; Fig. 3A). The overall nerve growth into the two compartments was comparable in size (550 μm OD), filled 73.8 ± 19.3% of the cross-sectional area (Fig. 3B), and elongated up to the agarose-capped end. Histological analysis confirmed the formation of perineurium and endoneurium in all groups. Qualitative evaluation of axonal composition in Toluidine-stained sections showed similar axonal growth among all groups. A cross-section of a nerve regenerated towards PTN (Fig. 3C) or BSA in the same Y-conduit is provided as a representative result (Fig. 3D).

**Figure 4:**
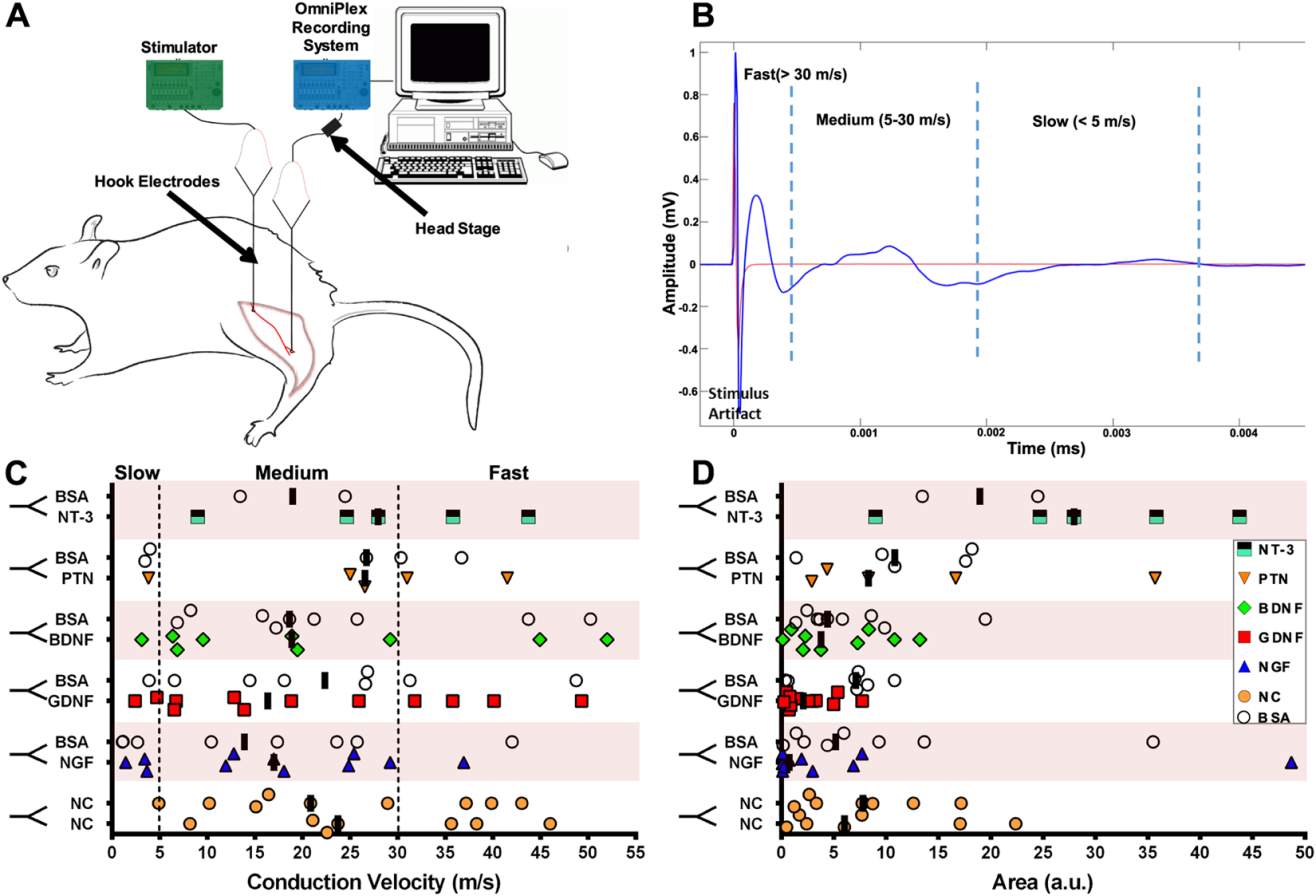
Regenerated split fascicles are electrically conductive. (**A**) Setup for measuring Compound Nerve Action Potential (CNAP). Bipolar hook electrodes provided stimulus pulses and the response recorded distally from the Y-split regenerated nerve fascicles. (**B**) Representative STA-CNAP waveform showing the stimulus artifact followed by fast, medium, and slow peak-responses. (**C**) Individual CNAP peaks with Conduction Velocity evoked by each treatment. The dotted lines indicate slow, medium, and fast responses from conducting fibers. (**D**) Corresponding individual area under the curve (AUC). Black bars represent the median evoked response per treatment. (N = 3–6 animals/group; data represents evoked CNAPs).

Ninety-seven percent of the split fascicles showed an evoked compound action potential, and only 3% had limited axonal growth and no CNAP response. Conduction velocity (CV) was calculated from the latencies of the peaks based on the distance from the stimulating and recording bipolar electrodes (Fig. 4A). Using 3X threshold potentials (1.5–3.0 V; Fig. 4B), slow (≤5 m/s), medium (5–30 m/s), and fast (>30 m/s) conducting CNAPs were evoked (Fig. 4C). The median responses were medium to fast in Y-tubes with GDNF (16.4 m/s), PTN (26.6 m/s), NT-3 (27.9 m/s), BDNF (18.9 m/s), and NGF (17.0 m/s), compared to nerve-caps (22.3 m/s) and averaged BSA (20.1 m/s). Quantification of the median peak areas showed small (NGF; 0.7 a.u.), medium (NT-3; 2.3, GDNF; 2.2, BDNF; 3.8 a.u.) and large (PTN; 6.0) peaks compared to nerve-caps (6.9 a.u.) and BSA (4.4 a.u.), demonstrating that the split fascicles are electrically conductive and providing some indication of the differential effect of the growth factors.

### GDNF increased the regeneration of both motor and sensory neurons, while NGF and PTN differentially influence sensory-axon regeneration

**Figure 5:**
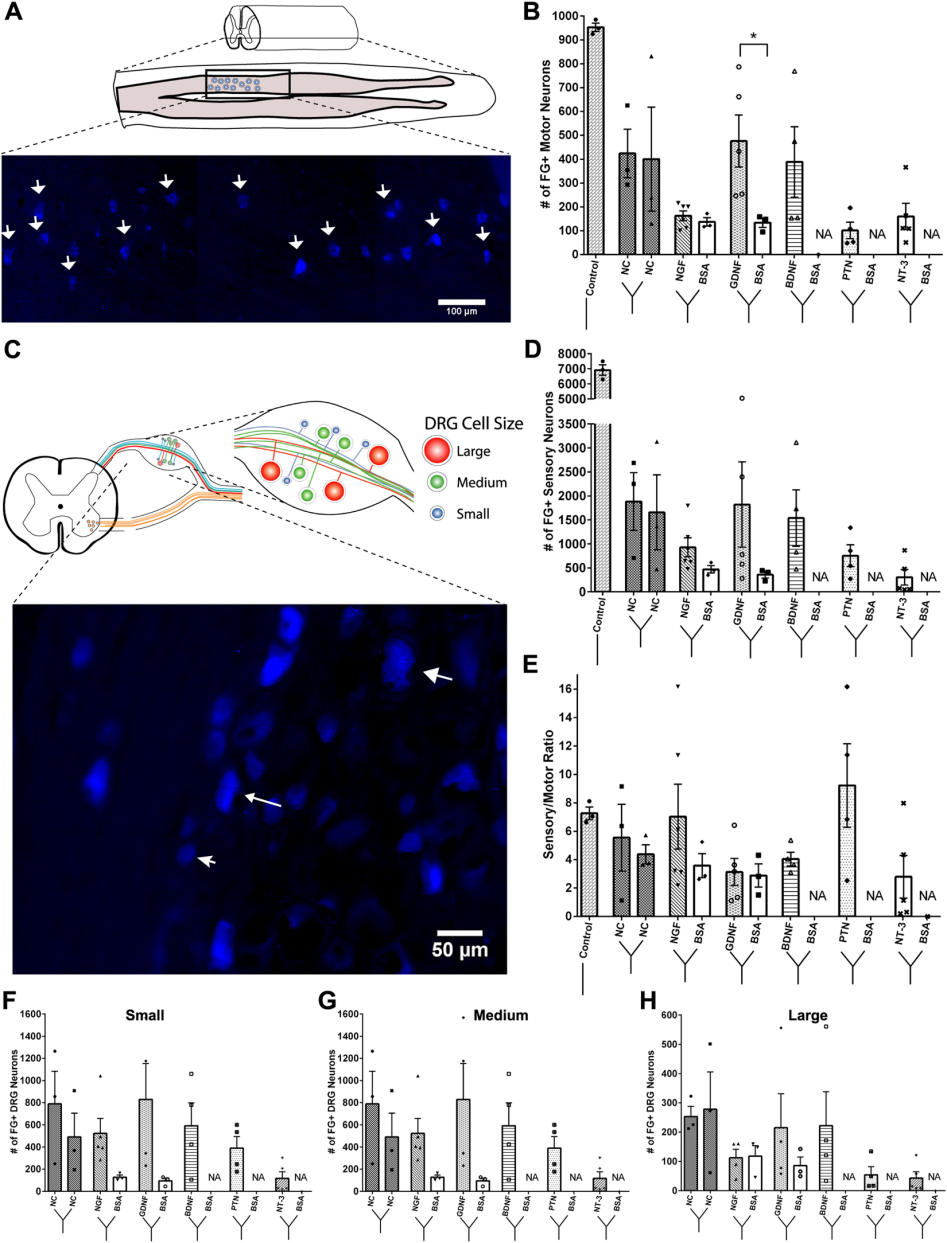
The effect of single guidance cues on the regeneration of motor and sensory neurons. (**A**) Schematic of the longitudinal section of the ventral spinal cord showing fluorogold positive cells (white arrows) (**B**) Quantification of FG+ cell in each of the split Y-nerve. GDNF had a significantly higher number of regenerated motor neurons compared to BSA. (**C**) Schematic of DRG soma size distribution and representative image of fluorogold positive cells of varying size. (**D**) Quantification of the total number of FG+ sensory neurons. (**E**) Single guidance cues did not significantly alter the sensory to motor ratio. (**F**–**H**) Distribution of small to large sensory neuron soma sizes showed no difference between the neurotrophic factor loaded microparticles and BSA loaded microparticles. Data represented as mean ± SEM. (*P ≤ 0.05). NA represents groups with failed retrograde tracing using FluoroRuby. (N = 3–6 animals/group).

The number of ventral motor neurons that regenerated into the separated Y-conduit compartments in non-injured controls was approximately 1000, i.e. 62.5% of the expected 1600 total population (Fig. 5A,B). Injured nerves attached to Y-conduits with nerve-caps as targets showed an even distribution of VMNs into both compartments (424 ± 176.4 and 400.3 ± 377.8). Conversely, when neurotrophic factors were used as distal targets, the different guidance cues significantly influenced the number of motor neurons innervating the two compartments (ANOVA: P ≤ 0.05; F (6, 19) = 8.824; R^2^ = 0.74). PTN (101.3 ± 34.38), NGF (162.5 ± 20.5), and NT-3 (160.0 ± 54.5) only attracted approximately 30% of the motor neurons. In sharp contrast, those growing into BDNF (388.5 ± 295.8), and GDNF (476.6 ± 242.6) compartments, were comparable to the nerve-caps. GDNF specifically attracted significantly more motor neurons than BSA in the other compartment of the same conduit (133.7 ± 33.9; P ≤ 0.05).

Quantification of the traced sensory neurons in the DRG of non-injured sciatic nerve controls showed 6914 ± 603.9 FG+ cells. This number was reduced and highly variable in the compartments of Y-conduits with nerve-cap targets (1883 ± 1042 and 1658 ± 1353; Fig. 5C,D). Both GDNF (1819 ± 1987) and BDNF (1540 ± 1176) attracted similar number of DRG sensory neurons compared to nerve-cap. These numbers were reduced in those growing towards NGF (929 ± 485.7), PTN (753.5 ± 456.9), and NT-3 (306.8 ± 362.9). To determine the effect of neurotrophic factors on sensory subtype regeneration, the DRG neurons were also evaluated based on perikaryal area (Fig. 5F–H). However, no statistically significant difference was observed between the small, medium, and large DRG neurons attracted by each of the neurotrophic factors loaded compartment.

The sensory-to-motor ratio for each type of treatment was estimated to be 7.3 ± 0.8 in non-injured sciatic nerves, which decreased slightly in Y-shaped conduits with nerve-cap controls (5.5 ± 4.0 and 4.4 ± 1.2) presumably due to the decreased sensory neuron regeneration following injury (Fig. 5E)^50^. The GDNF-compartment had the lowest sensory-to-motor ratio (3.1 ± 2.1) while the PTN compartment had the highest (9.2 ± 5.8) amongst all neurotrophic factor than BSA treatments and nerve-cap controls, but did not reach statistical significance. The sensory-to-motor ratios of compartments with NGF and GDNF, when compared to their respective BSA compartments, showed no significant effect. Together, these results suggest that specific neurotrophic factors can influence the axonal composition of split fascicles using a Y-conduit.

**Figure 6:**
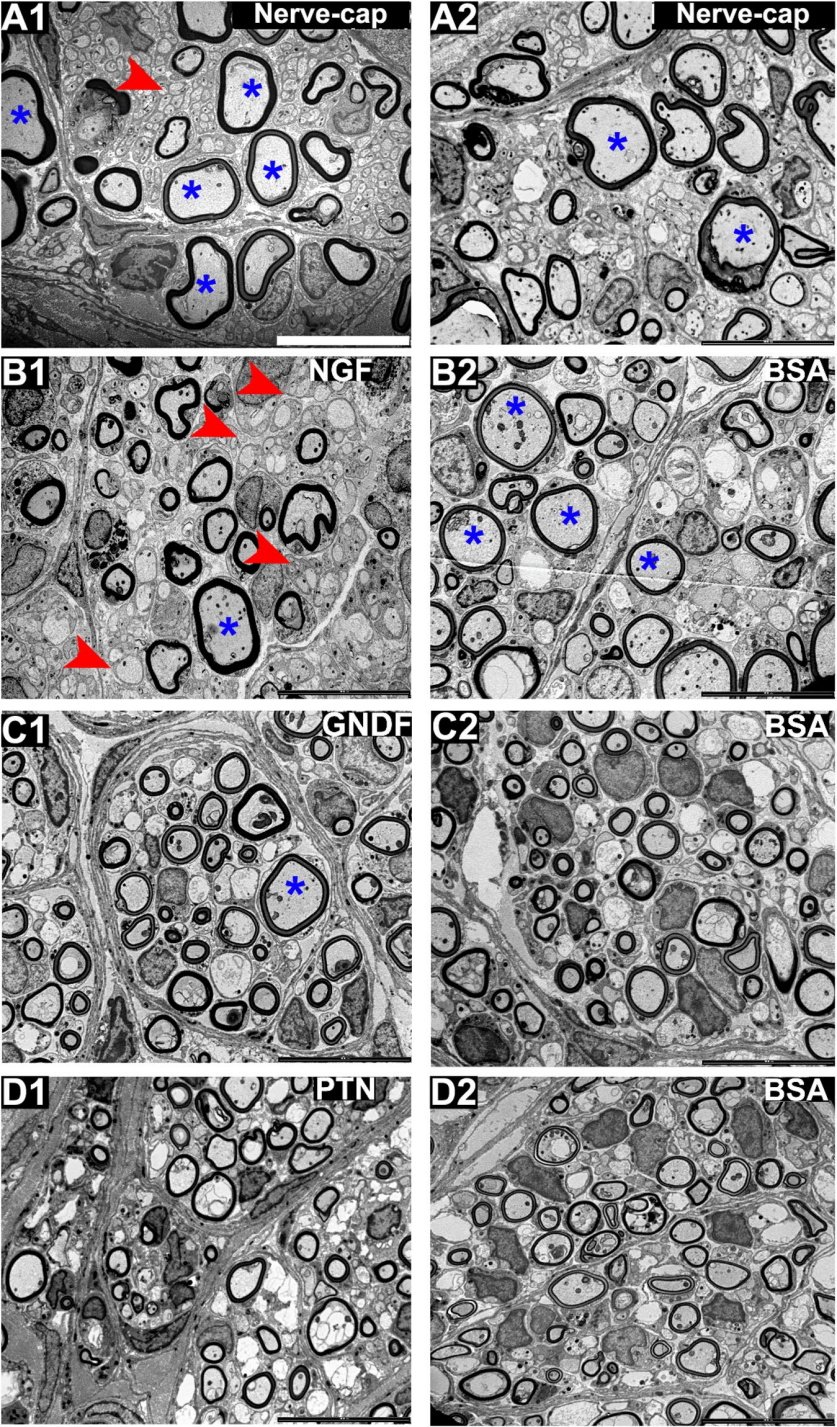
EM of the regenerated split Y-nerve fascicles showing normal axonal morphology with apparent large myelinated axons in the nerve-caps. Medium size axons in the GDNF, PTN, BSAs, whereas NGF shows higher density of unmyelinated axons. Large and unmyelinated axons are represented by * and (red arrow) respectively. Scale bar = 10 µm.

**Figure 7:**
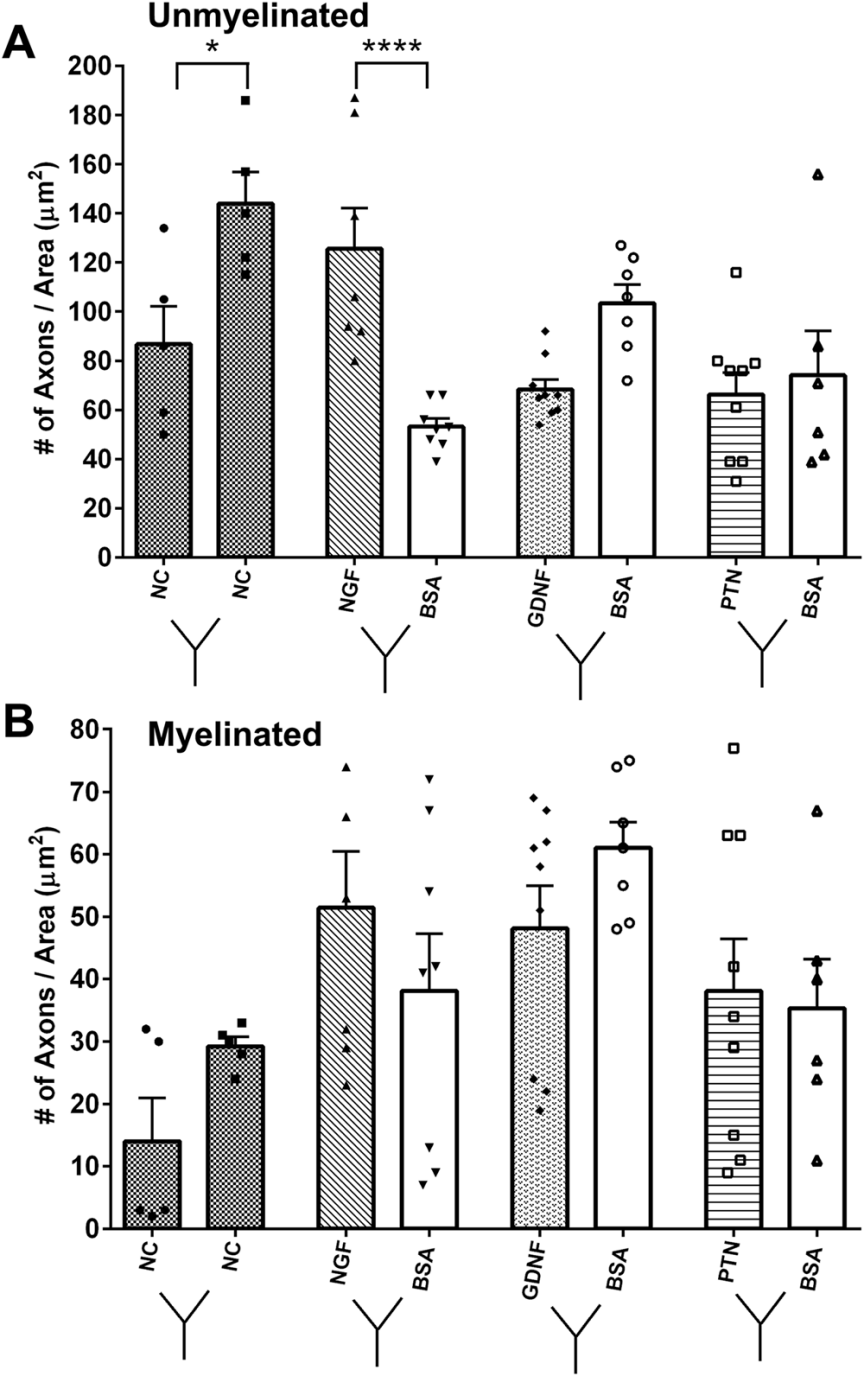
Influence of single NTFs on axon morphology for (**A**) Unmyelinated axonal and (**B**) myelinated axon count. Data represented as mean ± SEM. n = number of sampled EM pictures per group. (*P ≤ 0.05, ****P ≤ 0.0001).

Electron microscopy evaluation of the regenerated fascicles showed normal myelinated and unmyelinated axonal composition (Fig. 6) in all treatment groups. Large myelinated axons were evident in the nerve-caps and less abundant in the NTF treatments, and the myelin thickness appears to be similar among all the groups. The number of unmyelinated axons are more evident in the NGF treatment compared to the other NTF groups. The data implies that axonal composition is differentially affected by the NTF treatments. Quantification of the fibers types revealed a significant effect by the NTF treatment in unmyelinated axon count (one-way ANOVA; P ≤ 0.0001; F (7, 48) = 7.986; R^2^ = 0.538) (Fig. 7A). The NGF compartment (118.1 ± 44.1) had significantly higher number of unmyelinated axon count compared to BSA (66.3 ± 26.8). GDNF and PTN showed no difference in number of unmyelinated axon count when compared to their adjacent BSA compartment. The number of myelinated axons did not change significantly among the groups. Comparison of myelin thickness and fiber diameter between the experimental groups showed no difference (Supplementary Fig. S1).

### Specific guidance molecules can be used to differentially modulate the sensory and motor axons into separate regenerative chambers

**Figure 8:**
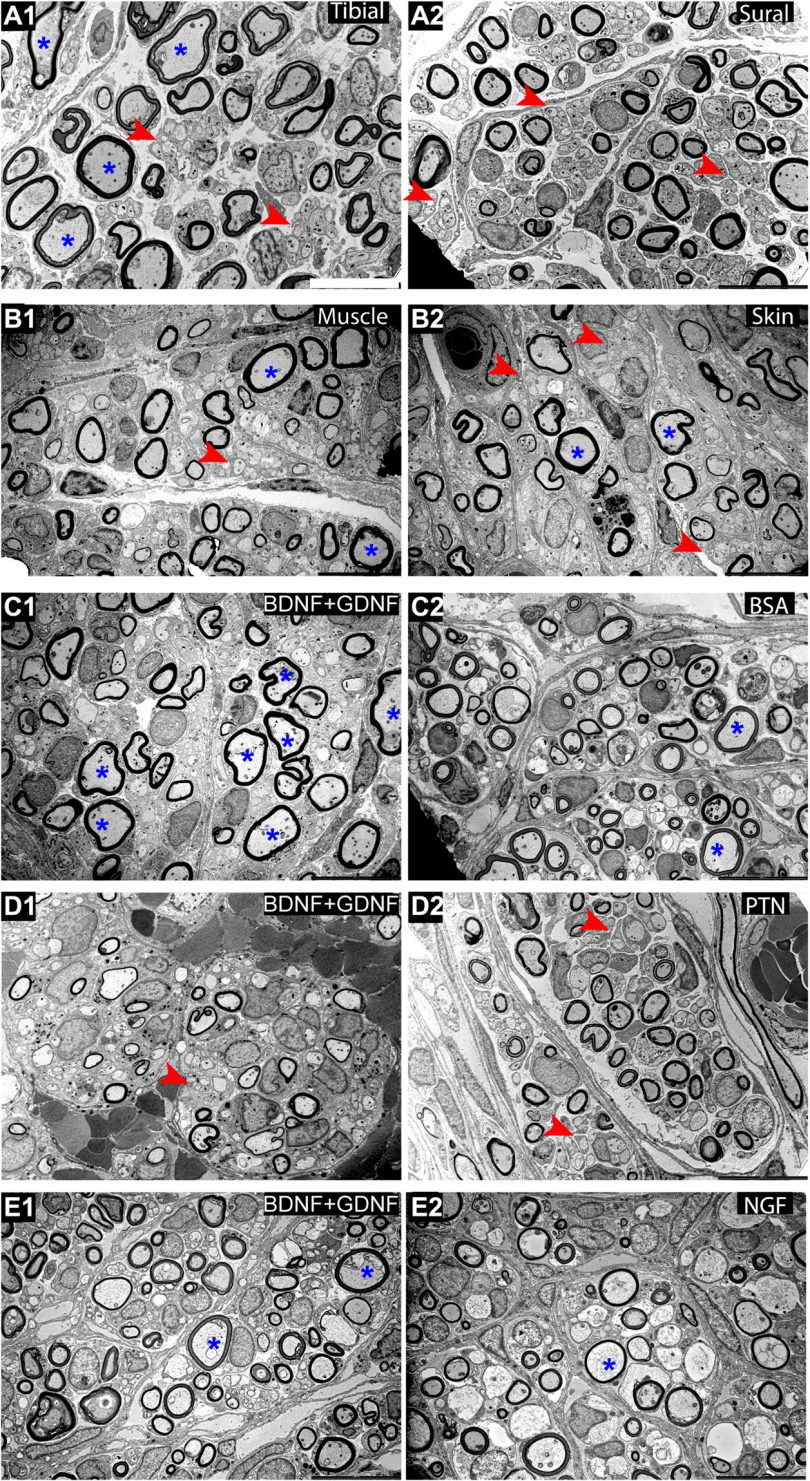
Representative SEM images of dual neurotrophic factors for motor (tibial, muscle-cap, and BDNF + GDNF) and sensory (sural, skin-cap, PTN, and NFG) targets showing normal axonal morphological composition. Large and unmyelinated axons are represented by * and (red arrow) respectively. Scale bar = 10 µm.

**Figure 9:**
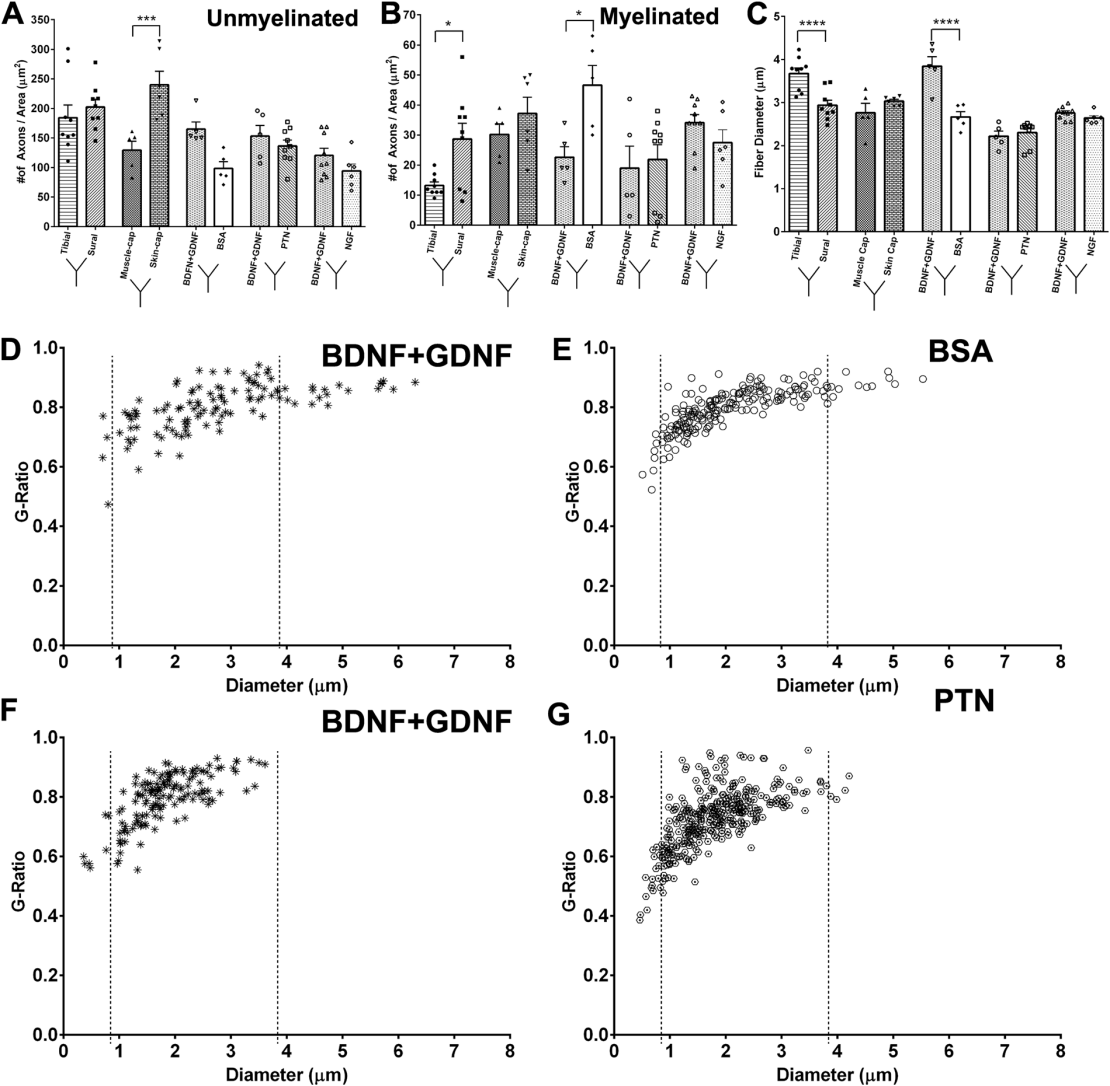
Morphological analysis of dual neurotrophic factors in split Y-nerve regeneration. (**A**,**B**) Quantification of the number of unmyelinated and myelinated axons. (**C**) Myelinated axon fiber diameter. (**D**–**F**) Scatter plots of G-ratio as a function of myelinated axon diameter; dotted lines represent distribution of small, medium, and large axons. Data represented at mean ± SEM. n = number of sampled EM pictures per group. (*P ≤ 0.05, ***P ≤ 0.001, ****P ≤ 0.0001).

Next, we tested the effect of presenting combination molecular guidance cues in separate compartments of the Y-choice assay (Fig. 8). As expected large myelinated axons were evident in the tibial and muscle-cap compartments compared to sural and skin-cap targets. The BDNF + GDNF when combined in one compartment and compared with BSA in the other showed a clear increase in the large myelinated axons. This effect was less evident when BDNF + GDNF was used against PTN and NGF in the other compartments. Quantification of axon morphology and composition revealed that the skin tissue had a significant effect using a one-way ANOVA (P ≤ 0.0001; F (9, 58) = 8.195; R^2^ = 0.560) (Fig. 9A). The regenerated nerve fascicle with the skin-cap showed significantly higher number of unmyelinated axon (240.0 ± 56.3) than the muscle-cap (123.4 ± 42.3). However, compartments with tibial-nerve or muscle-cap did not show the expected increase in the total number of myelinated axons (Fig. 9B). The number of myelinated axons in the sural-nerve (28.7 ± 15.7) and BSA (39.0 ± 7.6) compartment was significantly higher compared to the tibial-nerve (13.2 ± 3.5) and BDNF + GDNF (22.6 ± 7.9) compartment respectively (One-way ANOVA; P ≤ 0.01; F (9, 58) = 3.604; R^2^ = 0.36) (Fig. 9B). When fiber diameter was considered, there was a significant difference between the tibial- and sural-nerve group and the BDNF + GDNF and BSA compartment (One-way ANOVA; P ≤ 0.0001; F (9, 58) = 18.89; R^2^ = 0.75) (Fig. 9C). The fiber diameter of BDNF + GDNF (3.61 ± 0.78 µm) group was significantly higher compared to BSA (2.60 ± 0.57 µm) compartment, and comparable to the natural motor-related target of tibial-nerve (3.67 ± 0.38). The smaller diameter axons showed higher myelination with g-ratios 0.4 to 0.7, while the larger axons were thinly myelinated with g-ratios of 0.7 to 0.9. (Fig. 9D,E). Comparison of g-ratio and axon diameter of the BDNF + GDNF target with BSA and PTN revealed two distinct effects. A shift to the left in the myelinated axon diameter was observed in the BDNF + GDNF-PTN group (Fig. 9E,F) compared to the BDNF + GDNF-BSA (Fig. 9C,D).

**Figure 10:**
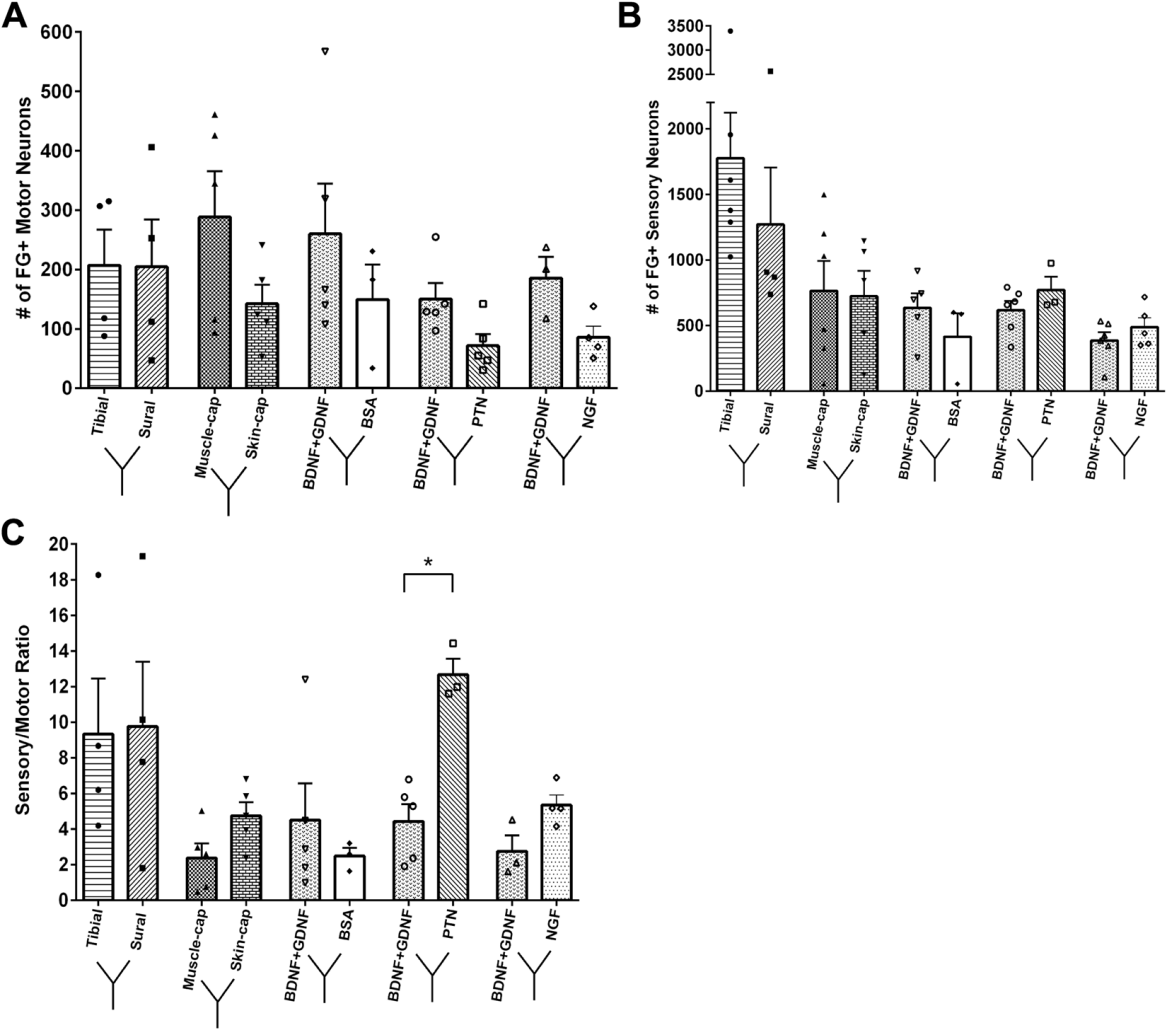
Effect of NTF combination on motor and sensory neuron regeneration. (**A**) FG+ motor neurons were quantified from the ventral spinal cord and (**B**) sensory neurons from the DRG. (**C**) The sensory to motor ratio of the FG+ cells. Data represented as mean ± SEM. (N = 3–6 animals/group; *P ≤ 0.05).

Retrograde labeling of the implants with Y-choice assay showed no specific preferential motor enrichment in the motor-mixed tibial nerve (207.0 ± 120.8 VMN) compared to the sensory sural nerve (204.5 ± 159.5) (Fig. 10A). In contrast, the muscle-cap attracted slightly more motor neurons (288.4 ± 172.8) when compared to the skin-cap (142.4 ± 71.67) without reaching statistical significance. The BDNF + GDNF compartments had increased number of motor neurons for all experimental groups versus PTN, NGF, or BSA control, however, the increase was not statistically significant by a one-ANOVA.

The total number of regenerated sensory neurons showed no significant difference among any of the treatments (Fig. 10B). A one-way ANOVA (P ≤ 0.05; F (9, 31) = 3.269; R^2^ = 0.49) revealed a significant effect in the comparison of sensory-to-motor (S/M) ratios across groups (Fig. 10C). The tibial-sural choice had comparable S/M ratios of 9.3 ± 3.1 and 9.7 ± 3.6 respectively. As expected the skin-cap and the NGF groups show an increased ratio compared to the muscle-cap and BDNF + GDNF counterpart, albeit not statistically significant. However, when BDNF + GDNF was presented along with PTN, the S/M ratio increased significantly in the PTN branch to 12.7 ± 0.9, suggesting this combination would be optimal in asymmetrically modulating the sensory to motor ratio.

## Discussion

Interfacing the damaged nerve in amputees in order to record motor intent and selectively stimulate distinct sensory-axons remains to be a significant challenge, despite the somatotopic organization in nerves^51^. Arguably, this is because most nerves are composed of a mixed number of motor, sensory, and autonomic axons. In the rat, the sciatic nerve has only 1,600 motor efferent axons from an approximated population of 27,000 axons, while 17,200 are sensory afferents and 8,200 are autonomic^47^. Thus, the probability of recording single units from motor axons (P = 0.059) or evoking specific sensory modalities is very low compared to that of interfacing with the large population of mixed sensory axons (P = 0.637). This study evaluated the possibility of using nerve growth factors to selectively attract specific subsets of neurons to distinct chambers to modulate the S/M ratio. Such modulated and guided axonal regrowth could improve the possibility of motor-intent recording and specific sensory stimulation.

Injured nerves are guided across an injury site by growth factors released by the distal nerve stump^52–54^. If a muscle or skin choice is present, spinal motor neurons will preferentially regenerate towards a muscle target. However, if the muscle target is unavailable, the motor neurons will grow towards the skin^55^. This path recognition is known to be mediated by growth factors upregulated by Schwann cells in the distal nerve stump^22,56^. Exogenous administration of these growth factors can be used to effectively guide the regenerating axons. NGF, when expressed in a distal cutaneous branch of the femoral nerve, selectively guides pain fibers across a bifurcation point and increases the S/M ratio^34^. This strategy has also proven effective in guiding injured adult pain fibers to re-innervate the spinal cord by expressing NGF in the dorsal horn after rhizotomy^57^. Our previous work extended these observations by demonstrating that injured axons can be guided to capped compartments in the arms of a Y-conduit, isolating the effects of neurotrophic factors to the growing axons^40^. In that study, we showed that compartmentalized diffusion of NGF and NT-3 differentially directed the regeneration CGRP + nociceptive and N-52 + large diameter axons (both sensory and motor).

Here, we further demonstrate that using exogenous neurotrophic factors *in vivo*, the mixed rat sciatic nerve can be differentially guided into functional fascicles with normal microanatomy. When nerve-cap control segments were used distally in the arms of the Y-conduit, the number of motor neurons divided efficiently and symmetrically into two fascicles each with 50% of the motor and 29% of the sensory neurons compared to the total population in non-injured animals. The disproportional reduction in the number of DRG neurons compared to the uninjured controls is in agreement with expected cell death in this population after injury^50^. The addition of GDNF into one of the compartments mediated the regeneration of motor population to levels comparable to the nerve-cap and increased the number of sensory neurons. The effect on the sensory axons can be attributed to the increased expression of GDNF receptors GFRα-1 and Ret in the large diameter neurons, and of GFRα-3 in the small diameter sensory neurons after injury^58^. Compared to BSA compartments, GDNF significantly increased the total number of motor neurons 3.7 fold, confirming the its ability to modulate the motor content of regenerated nerves. In contrast, neither NGF and PTN showed a significant effect on attracting motor neurons.

With respect to sensory axons, NGF was more effective in enticing nerve growth, as the number of unmyelinated axons in the regenerated fascicle was significantly increased compared to PTN or GDNF. This effect was specific, and is consistent with axonal sprouting rather than axonal guidance, which has been recognized in injured nerves^59^. The observation that PTN, a known motor neuron growth factor^22,39^, did not influence the number of motor neurons, but rather significantly increase the S/M ratio was unexpected. However, PTN is known to be up-regulated in the DRG satellite cells, Schwann cells, macrophages, and endothelial cells, which express the anaplastic lymphoma kinase (ALK) PTN receptor in the distal portion of the nerve after injury^22^. It is also reported that TrkA + nociceptor neurons express the ALK receptor^60^. Moreover, PTN can significantly enhance regeneration of myelinated axons across a nerve graft in adult rats, and this effect can be blocked by an ALK antibody, suggesting that ALK is the receptor responsible for PTN’s neurotrophic activity^39^. Together, these results provide support for the use of GDNF in modulating the number of motor axons and of PTN in influencing sensory neurons in their path for separate compartments.

Evaluation of motor neuron regeneration using two different target tissues in  the Y-choice assay revealed that the tibial-sural and muscle-skin-cap are comparable. However, in the NTF groups, BDNF + GDNF doubled the number of motor neurons compared to those growing into PTN and NGF compartments. Consistently, the fiber diameter was found to be significantly larger in the BDNF + GDNF compared to BSA. Conversely, the S/M ratio was significantly increased in the PTN compartment. Previously, the BDNF + GDNF combination has been used in other studies to increase the number of regenerated motor neurons^61^. However, this is the first study to show an *in vivo* Y-choice assay between BDNF + GDNF and PTN to differentially modulate the axonal content in those chambers. We noted that the total number of neurons growing into the different compartments using multiple growth factors was reduced approximately by 60% compared to the single growth factor treatments. We interpret this as an indication that the higher concentration of microparticles might have presented a physical barrier for nerve regeneration. We have recently developed a method to produce sustained gradients using polymeric coils in the lumen of micro channels to address this issue^43^. Future studies will implement NTFs delivery methods with open lumen for maximal nerve growth.

While this study was successful in modulating the sensory-to-motor ratio of neurons in specific compartments, we had expected NGF, BDNF, and NT-3 to increase the regeneration of small, medium, and larger diameter sensory neurons respectively. However, the level of resolution on retrograde-traced sensory subtypes was not definitive. In our samples, FG masked the calcitonin gene-related peptide (CGRP) marker and thus confirmation of specific sensory subtypes was not obtained. Ideally, molecular guidance cues would be utilized to differentially control the number of rapid adapting (RA) and slow adapting (SA) fibers in a Y-choice nerve conduit in order to encode for specific functions such as form, texture, grip control/motion detection, tool manipulation, and hand conformation^62^. Given the results in this study, the goal of achieving regenerative control of mechanoceptive fibers seems feasible, but not trivial. It is possible that the addition of selective molecular inhibitors could improve this current strategy. Molecular repellents play a crucial role in creating patterns of somatosensory innervation by restricting growth cone extension and branching^63^. Chemorepellents such as Semaphorins are known to restrict the path of nociceptive regenerating axons both during development and in the adult spinal cord^64–66^. Recently, the combination of NGF and Sema3A was used in the spinal cord, where NGF attracted TrkA + pain fibers into the dorsal horn after rhizotomy, and simultaneous expression of Sema3A in the ventral horn restricted neurite extension into that area^67^. Strategies combining attractive and repulsive cues therefore could be utilized to refine axon guidance into separate recording and stimulating compartments. In addition to NTFs, neural cell adhesion molecules such as the polysialic acid, N-CAM, L1 and N-cadherin are known to be essential for proper motor axon guidance^68^.

Together this data demonstrates that transected mixed nerves can be regenerated to form fascicles with differential content of motor and sensory axons. However, several limitations remain: First, evaluations were conducted after 45-days and longer studies are needed to confirm axonal stability over time. Second, future studies should include the testing of Y-shaped REMIs, equipped with multi-electrode arrays to confirm the advantage of molecular guidance in increasing the probability of recording from motor axons and evoking specific sensations from regenerated sensory fascicles. Third, this study was performed in rodents, and given the anatomical differences with higher vertebrate species, further testing is needed to confirm that similar results can be obtained in non-human primates and fully determine the feasibility of this molecular guidance approach for peripheral neural interfacing.

In summary, molecular guidance offers a new approach, which can be added to a comprehensive strategy to obtain directed axonal regeneration in nerve repair, or as a method to influence the axon type content in regenerative neural interfacing.

## Electronic supplementary material

Supplementary Figure S1
