# Supplementary material for: Asymmetric Sensory-Motor Regeneration of Transected Peripheral Nerves Using Molecular Guidance Cues
Source: Sci Rep. 2017 Oct 30;7:14323. doi: 10.1038/s41598-017-14331-x (PMC5662603; doi:10.1038/s41598-017-14331-x)
Supplement: Supplementary file 1 — Supplementary Figure S1 [file 41598_2017_14331_MOESM1_ESM.pdf]

Asymmetric Sensory-Motor Regeneration of Transected Peripheral Nerves Using Molecular Guidance Cues

Sanjay Anand[1], Vidhi Desai[2], Nesreen Alsmadi[1], Aswini Kanneganti[1], Dianna Huyen-Tram Nguyen[2], Martin Tran[2], Lokesh Patil[1], Srikanth Vasudevan[5], Cancan Xu[2], Yi Hong[2], Jonathan Cheng[3], Edward Keefer[4] and Mario I. Romero-Ortega[1*]

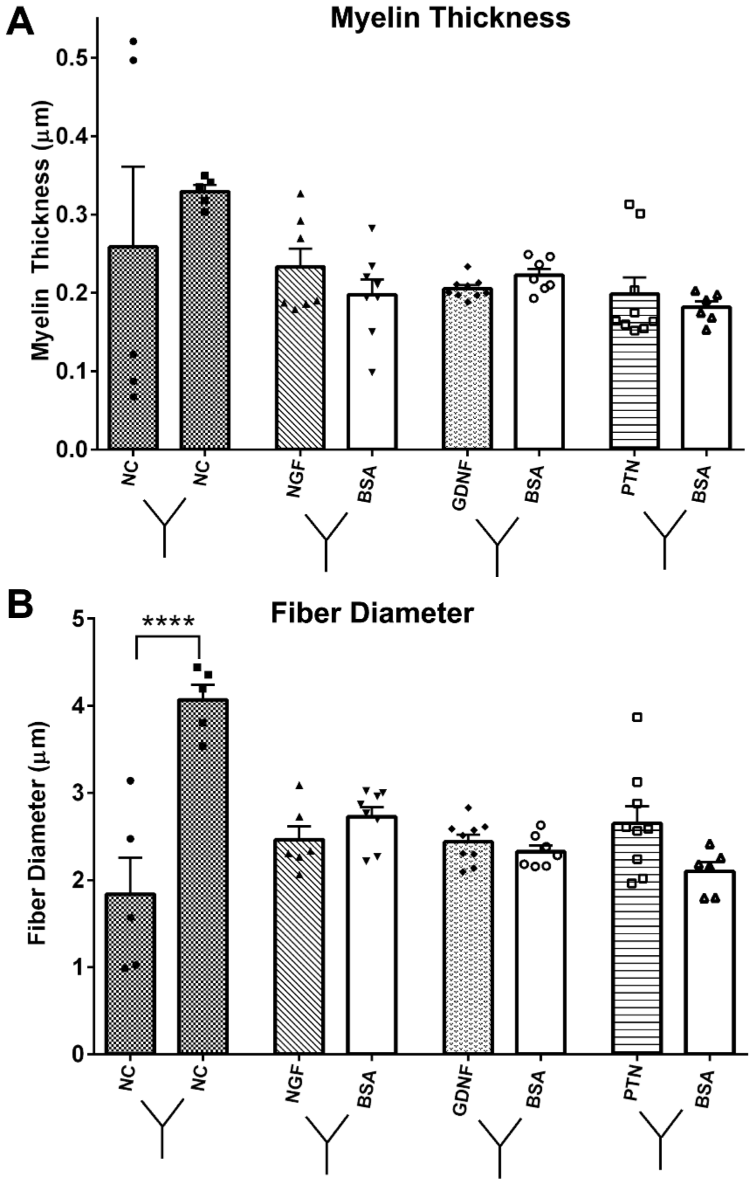

Supplementary Figure S1: NTFs effect on (A) myelin thickness and (B) Fiber diameter when compared to BSA. n = number of sampled EM pictures per group. **** P ≤ 0.0001.
